# Impaired neutralizing antibody responses against the BA1.1, BA.2, and BA.5 Omicron SARS-CoV-2 subvariants in hospitalized adults infected with pre-Omicron variants in Argentina

**DOI:** 10.1128/spectrum.03479-25

**Published:** 2026-01-21

**Authors:** J. Sachithanandham, I. A. Sitaras, R. A. Albertini, R. H. Capra, J. Marquez, G. B. Sadino-Vallve, P. R. Gargantini, P. R. Cortes, N. E. Ponce, A. van de Guchte, A. S. Gonzalez-Reiche, N. B. Olivero, V. E. Zappia, M. Hernandez-Morfa, A. Echenique-Nunez, M. Nunez-Fernandez, L. Raya-Plasencia, H. van Bakel, A. Pekosz, D. R. Perez, J. Echenique

**Affiliations:** 1 W. Harry Feinstone Department of Molecular Microbiology and Immunology, Johns Hopkins Bloomberg School of Public Health 25802 , Baltimore, Maryland, USA; 2 Instituto Universitario de Ciencias Biomédicas de Córdoba (IUCBC) 602578, Córdoba, Argentina; 3 Servicio de Clínica Médica, Hospital Privado Universitario de Córdoba 62998 , Córdoba, Argentina; 4 Servicio de Laboratorios, Hospital Privado Universitario de Córdoba 62998 , Córdoba, Argentina; 5 Clínica Universitaria Reina Fabiola, Universidad Católica de Córdoba 28187, Córdoba, Argentina; 6 Centro de Investigaciones en Bioquímica Clínica e Inmunología (CIBICI)-Consejo Nacional de Investigaciones Científicas y Técnicas (CONICET), Córdoba, Argentina; 7 Departamento de Bioquímica Clínica, Facultad de Ciencias Químicas, Universidad Nacional de Córdoba 28217, Córdoba, Argentina; 8 Department of Genetics and Genomics Sciences, Icahn School of Medicine at Mount Sinai 5925, New York, New York, USA; 9 Centro de Química Aplicada, Facultad de Ciencias Químicas, Universidad Nacional de Córdoba 28217, Córdoba, Argentina; 10 Department of Pathology, Molecular, and Cell-Based Medicine, Icahn School of Medicine at Mount Sinai 5925, New York, New York, USA; 11 Icahn Genomics Institute, Icahn School of Medicine at Mount Sinai 5925, New York, New York, USA; 12 Department of Population Health, College of Veterinary Medicine, University of Georgia 1355, Athens, Georgia, USA; Emory University School of Medicine, Atlanta, Georgia, USA

**Keywords:** SARS-CoV-2, Omicron, variants of concern, antibody, pre-Omicron, Delta, Gamma, neutralizing antibodies, vaccine, comorbidities

## Abstract

Infection with SARS-CoV-2 variants typically confers protective humoral immunity. Yet, the Omicron variant demonstrates a considerable capacity to evade immune responses in individuals previously infected with other SARS-CoV-2 strains. This has prompted investigation into Omicron’s potential resistance to neutralizing antibodies elicited by natural infection or vaccination. Importantly, the molecular mechanisms underlying this cross-protection have been investigated; however, they are not yet fully elucidated. In this study, we investigated the neutralization capacity of convalescent sera from COVID-19 patients in Córdoba City, Argentina, offering insights into immune responses against various SARS-CoV-2 variants. We analyzed samples from hospitalized patients infected with the Lambda and Gamma variants during the pandemic’s second wave from May to August 2021. These patients showed strong neutralization against Gamma, moderate activity against the B.1 and Delta variants, a weak response to Omicron BA.2, and extremely poor cross-neutralization against the Omicron BA.1.1 and BA.5 subvariants. Additionally, we examined convalescent sera from ambulatory patients during the fourth COVID-19 wave, from May to July 2022, when various Omicron variants predominated. Consistent with the hospitalized cohort, the highest neutralizing activity was observed against the Gamma variant, with moderate responses against B.1 and Delta, and low activity against Omicron subvariants. These findings highlight the critical need for region-specific studies of neutralizing responses to inform the development of future SARS-CoV-2 vaccine formulations.

**IMPORTANCE:**

Investigating the neutralizing capacity of sera obtained from COVID-19 patients infected with distinct viral variants—in this case, a comparison between pre-Omicron and Omicron strains—is essential for elucidating mechanisms of immune evasion, cross-protective immunity, and the efficacy of vaccines. Variations in neutralization profiles provide critical insights that inform the development of updated vaccines and booster immunization strategies, particularly given the pronounced immune escape characteristics exhibited by the Omicron variant. Furthermore, it is imperative to examine geographic heterogeneity, as regional differences in variant prevalence, vaccine types, and public health interventions contribute to diverse immune profiles. Conducting such investigations is critical for informing the development of tailored public health policies, facilitating the identification of immunological susceptibilities and resilience within distinct populations. This understanding is essential for improving preparedness in response to the emergence of new SARS-CoV-2 variants.

## INTRODUCTION

Since its emergence in 2019, the COVID-19 pandemic has been shaped by the continuous evolution of the SARS-CoV-2 virus. Through a process of mutation and natural selection, the viral genome has acquired changes that provide a survival advantage, leading to the development of variants of concern (VOCs). These VOCs exhibit enhanced characteristics, such as increased transmissibility, improved cellular infectivity, and greater ability to evade the immune system, collectively contributing to the ongoing challenges in controlling the disease (1). Argentina’s experience with the COVID-19 pandemic mirrored global trends, exhibiting distinct waves with fluctuating severity. A notable shift occurred with the third and fourth waves, commencing in November 2021. These surges were primarily fueled by the rapid emergence and dominance of the Omicron variant, which swiftly outcompeted the previously prevalent Delta variant. This transition led to an unprecedented surge in both infections and hospitalizations, surpassing the peaks observed during earlier waves driven by Alpha, Beta, and Delta variants (2). Between November 2021 and July 2022, the Omicron variant of SARS-CoV-2 underwent significant evolution, giving rise to several subvariants such as BA.1, BA.2, and BA.5. These subvariants rapidly achieved dominance in numerous countries globally, including Argentina (3, 4).

As the COVID-19 pandemic progressed, the SARS-CoV-2 virus faced mounting and significant immune pressure, which likely drove the selection of mutations in areas targeted by neutralizing antibodies (Nabs) (5). These changes primarily occurred in the receptor-binding domain and the N-terminal domain of the virus’s Spike glycoprotein. Such modifications enhanced the virus’s ability to evade the host immune response, leading to considerable concern about immune escape (6).

The Omicron variant of SARS-CoV-2 significantly evades polyclonal neutralizing antibodies from prior infection or vaccination, leading to reduced vaccine effectiveness against its subvariants (3). Studies have shown that serum from individuals infected with pre-Omicron variants produced varying levels of neutralizing antibodies against Omicron, ranging from high (7) to moderate (8) and often low (9–11). These findings highlight the critical need not only for epidemiological surveillance but also for routine measurement of Nab titers in populations exposed to new SARS-CoV-2 variants or subvariants. Such research is crucial for developing effective vaccination strategies (3).

This study investigated the neutralizing antibody responses to various SARS-CoV-2 variants, including the ancestral B.1 as well as the Gamma, Delta, and Omicron-derived BA.1.1, BA.2, and BA.5 strains. We compared responses in two groups: unvaccinated adults infected during the pre-Omicron phase and vaccinated adults with natural Omicron subvariant infections. Our analysis revealed that individuals previously infected with the Gamma and Lambda variants showed a significantly diminished immune response against Omicron subvariants, especially BA.1.1 and BA.5. These findings underscore the importance of assessing region-specific neutralizing antibody profiles, as these are shaped by diverse local factors. Such data are important for informing the development of future vaccine strategies.

## RESULTS

### Patient cohorts and demographics and clinical presentation of COVID-19 patients

This study analyzed two distinct cohorts of adult COVID-19 patients from Córdoba City, Argentina. The first cohort (*n* = 20), hospitalized between May and August 2021, comprised individuals infected with pre-Omicron variants (predominantly Gamma and Delta), half of whom had received only a single SARS-CoV-2 vaccine dose. This cohort (12 males, 8 females; median age 48, range 28–69 years old) was recruited from Private University Hospital of Córdoba, Argentina (HPU; https://hospitalprivado.com.ar). Initial diagnoses for the first cohort were made via immunochromatography-based antigenic assays, with subsequent confirmation by quantitative reverse transcription polymerase chain reaction. RNA extracted from nasopharyngeal swab samples of these patients underwent genome sequencing to identify SARS-CoV-2 variants, following established protocols (12). Analysis revealed that the Gamma (70%) and Lambda (25%) variants predominated among the patient population (Table 1). Upon hospital admission, all 20 patients presented with respiratory symptoms, and viral pneumonia was diagnosed in 16 individuals (80%), characterized by bilateral opacities on chest radiographs. Per WHO guidelines (13), 50% of patients (10/20) were classified as severe, 20% (4/20) as moderate, and 30% (6/20) as mild. The number of severe cases was significantly higher than moderate cases (*P* = 0.0495) but not significantly different from mild cases. Among severe cases (ages 31–65, average 54.1 years), significantly more males than females were under 60 years old (*P* = 0.0014). When severe and moderate cases were combined, a statistically significant difference was observed (*P* = 0.0125) compared to the mild cases (Table 1). Most patients (17/20, 85%) had at least one comorbidity, with 5 (25%) having two and 4 (20%) having three. Obesity was the most common comorbidity (9/20, 45%), followed by hypertension (5/20, 25%), dyslipidemia (4/20, 25%), diabetes (3/20, 15%), and pregnancy (3/20, 15%). Renal, respiratory, and cardiac diseases were less frequent (2/20, 10%) (Table 1).

**TABLE 1:** Characteristics of the first (Hospital Privado Universitario de Córdoba, May–August 2021) and the second (Clinica Universitaria Reina Fabiola, May–July 2022) patient cohorts selected for the evaluation of cross-neutralization activity

Characteristic	First patient cohort			Second patient cohort (vaccinated; *n* = 12)
	Unvaccinated (*n* = 10)	Vaccinated (*n* = 10)	All (*n* = 20)	
Age (years), median (range)	45.1 (28–67)	50.8 (24–69)	47.9 (24–69)	32.7 (24–51)
<30, *n* (%)	1 (10)	1 (10)	2 (10)	1 (8.3)
30–39, *n* (%)	4 (40)	1 (10)	5 (25)	1 (8.3)
40–49, *n* (%)	2 (20)	2 (20)	4 (20)	4 (33.3)
50–59, *n* (%)		5 (50)	5 (25)	2 (16.6)
60–69, *n* (%)	3 (30)	1 (10)	4 (20)	2 (16.6)
70–79, *n* (%)				2 (16.6)
Gender, *n* (%)				
Female	6 (60)	2 (20)	8 (40)	6 (50)
Male	4 (40)	8 (80)	12 (60)	6 (50)
Severity of illness, *n* (%)				
Asymptomatic				
Symptomatic	10 (100)	10 (100)	20 (100)	12 (100)
Pneumonia	9 (90)	7 (70)	16 (80)	
Mild	3 (30)	3 (30)	6 (30)	12 (100)
Moderate	3 (30)	1 (10)	4 (20)	
Severe	4 (40)	6 (60)	10 (50)	
Ambulatory				12 (100)
Hospitalization	10 (100)	10 (100)	20 (100)	
ICU admission				
Comorbidities, *n* (%)				
None		3 (30)	3 (15)	1 (8.3)
Obesity	6 (60)	3 (30)	9 (45)	6 (50)
Pregnancy	3 (30)		3 (15)	
Hypertension	1 (10)	4 (40)	5 (25)	5 (41.6)
Diabetes	2 (20)	1 (10)	3 (15)	2 (16.6)
Dyslipidemia	2 (20)	2 (20)	4 (20)	4 (33.3)
Respiratory diseases		2 (20)	2 (10)	2 (16.6)
Renal diseases		2 (20)	2 (10)	
Cardiac diseases	1 (10)	1 (10)	2 (10)	1 (8.3)
Hepatic diseases				1 (8.3)
Autoimmune diseases		1 (10)	1 (5)	
Hypothyroidism				7 (58.3)
Cancer				1 (8.3)
Vaccination, *n* (%)				
First dose		10 (100)	10 (50)	
Third/fourth doses				12 (100)
SARS-CoV-2 variants (%)				
Gamma			70	
Lambda			25	
Omicron BA.2				15
Omicron BA.2.1				5
Omicron BA.2.3				40
Omicron BA.2.9				30
Omicron BA.5				10

Between May and July 2022, a second cohort of 12 ambulatory adult patients with mild COVID-19 was recruited from Reina Fabiola’s University Clinic (CURF). All patients, equally split between sexes (six females, six males; median age 32.7, range 24–51), had received at least three vaccine doses and likely had prior SARS-CoV-2 infections, with diagnoses confirmed by PCR assays. In this cohort, 4/12 (33.3%), 1/12 patients (8.3%), and 6/12 (50%) patients presented with one, two, and three comorbidities, respectively. Hypothyroidism was significantly more prevalent in women (58.3%) compared to men (*P* = 0.0279). Additional comorbidities were observed: obesity was the most prevalent, affecting 50% (*n* = 6) of participants, followed by hypertension at 41.6% (*n* = 5) and dyslipidemia at 33.3% (*n* = 4). Other observed comorbidities included diabetes mellitus and respiratory diseases, both at 16.6% (*n* = 2) each. Cardiac, hepatic, and cancer diagnoses were each present in 8.3% (*n* = 1) of the participants. Due to logistical constraints, nasopharyngeal samples were not collected from this specific cohort. However, sequencing of 20 concurrent samples (Table 1) from other ambulatory COVID-19 patients at CURF confirmed the circulation of various Omicron subvariants during the same period, including BA.2 (15%), BA.2.1 (5%), BA.2.3 (40%), BA.2.9 (30%), and BA.5 (10%). These sets of samples were sequenced using Nanopore technology, as previously described (4).

### Diminished Omicron neutralization after pre-Omicron SARS-CoV-2 infection in hospitalized adults in Córdoba, Argentina

Given the rise in Omicron infections, even among vaccinated individuals, our goal was to investigate the neutralizing capacity developed from prior infections or vaccinations. We analyzed serum samples from COVID-19 patients infected with pre-Omicron variants between May and August 2021 (first cohort). Samples were collected both in the early stages of infection and again 30 days later during the convalescent phase.

**Fig 1:**
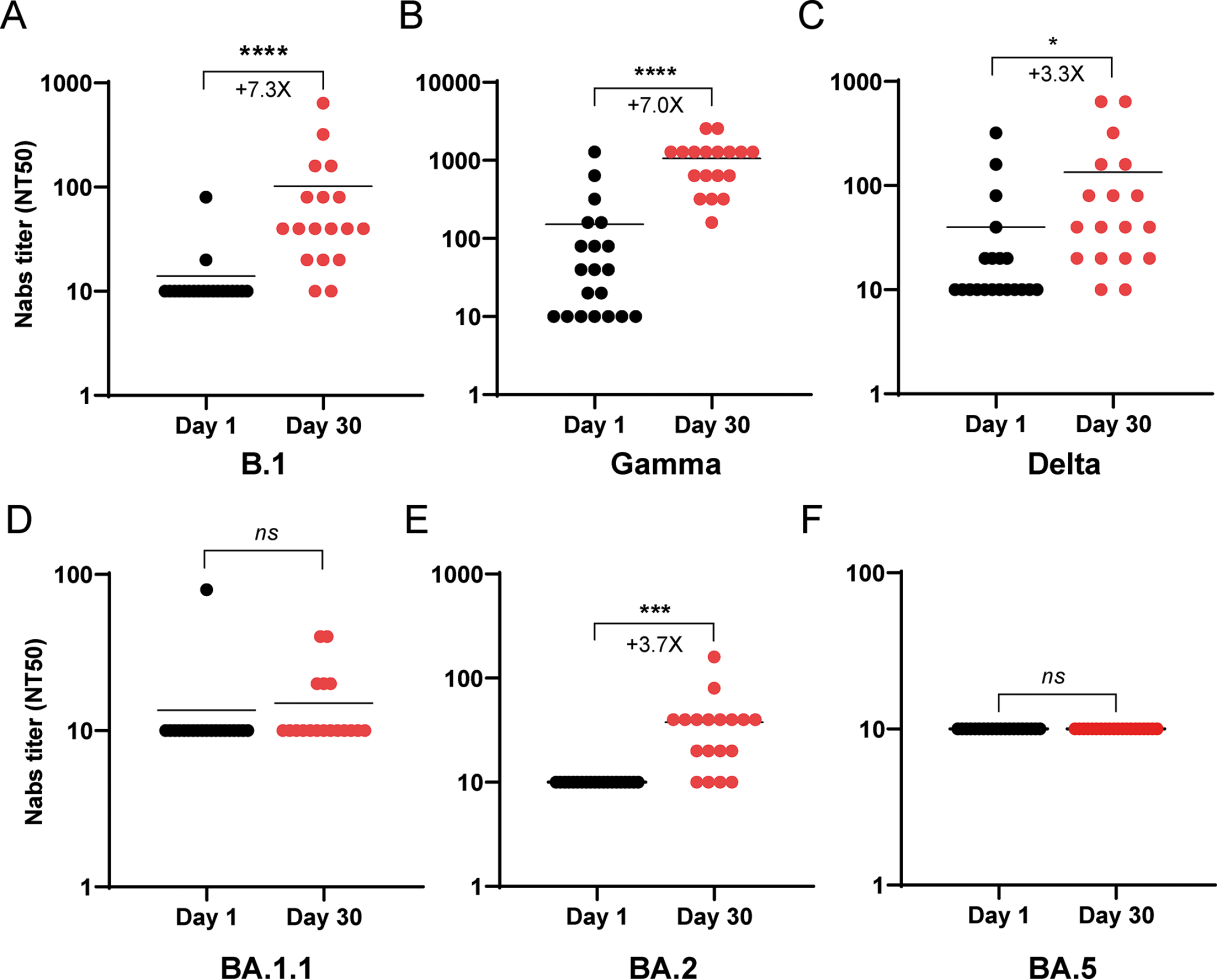
Determination of serum neutralizing activity in hospitalized COVID-19 patients recruited from May to August 2021 (cohort 1). The Nab titers were tested against pre-Omicron strains, including B.1 (**A**), Gamma (**B**), and Delta (**C**), as well as Omicron variants, such as BA.1.1 (**D**), BA.2 (**E**), and BA.5 (**F**). Results are expressed as NT50 values on a logarithmic scale. Each circle represents an individual patient; black circles correspond to sera collected on day 1 post-hospitalization, while red circles represent sera collected on day 30 during the convalescent stage. The fold change of the mean Nab titers and significant *P* values are indicated below and on the line, respectively. Asterisks denote statistically significant differences between sample groups (*, *P* < 0.05; ***, *P* < 0.001; ****, *P* < 0.0001; ns, not significant).

Sera collected from hospitalized patients on day 1 were analyzed for their neutralizing efficacy against a panel of SARS-CoV-2 variants, encompassing pre-Omicron strains (B.1 [D614G], Gamma, and Delta) and Omicron sublineages (BA.1.1, BA.2, and BA.5). A clear differential in neutralizing activity was observed. The acute-phase sera collected at day 1 demonstrated variable neutralization against pre-Omicron variants. Specifically, 5.5% of samples neutralized B.1; 61% neutralized Gamma; and 44.4% neutralized Delta (Fig. 1A through C). The same sera showed no detectable neutralizing activity against any of the tested Omicron isolates (Fig. 1D through F). This highlights a significant escape of Omicron variants from the humoral immune responses elicited during initial SARS-CoV-2 infections. Analysis of convalescent serum samples, collected on day 30 from the same patient cohort, revealed a significant increase in NAb titers in most samples compared to acute-phase samples collected on day 1 (see Fig. 1A and B). This increase was particularly pronounced against the B.1 and Gamma variants. The highest NAb titers were observed against the Gamma variant (Fig. 1B), which is consistent with the patients’ history of infection with either the Gamma or Lambda variants. The convalescent sera had variable ability to recognize Omicron variants, with NAb titers against the BA.1.1 and BA.5 subvariants not showing a statistically significant increase from days 1 to 30, while a 3.3-fold increase was noted for the BA.2 subvariant in NAb titers at day 30. The overall levels of NAbs directed against the Omicron subvariants remained significantly lower than those observed for pre-Omicron strains, given the large number of convalescent sera samples that did not recognize the Omicron variants (see Fig. 1D through F).

**Fig 2:**
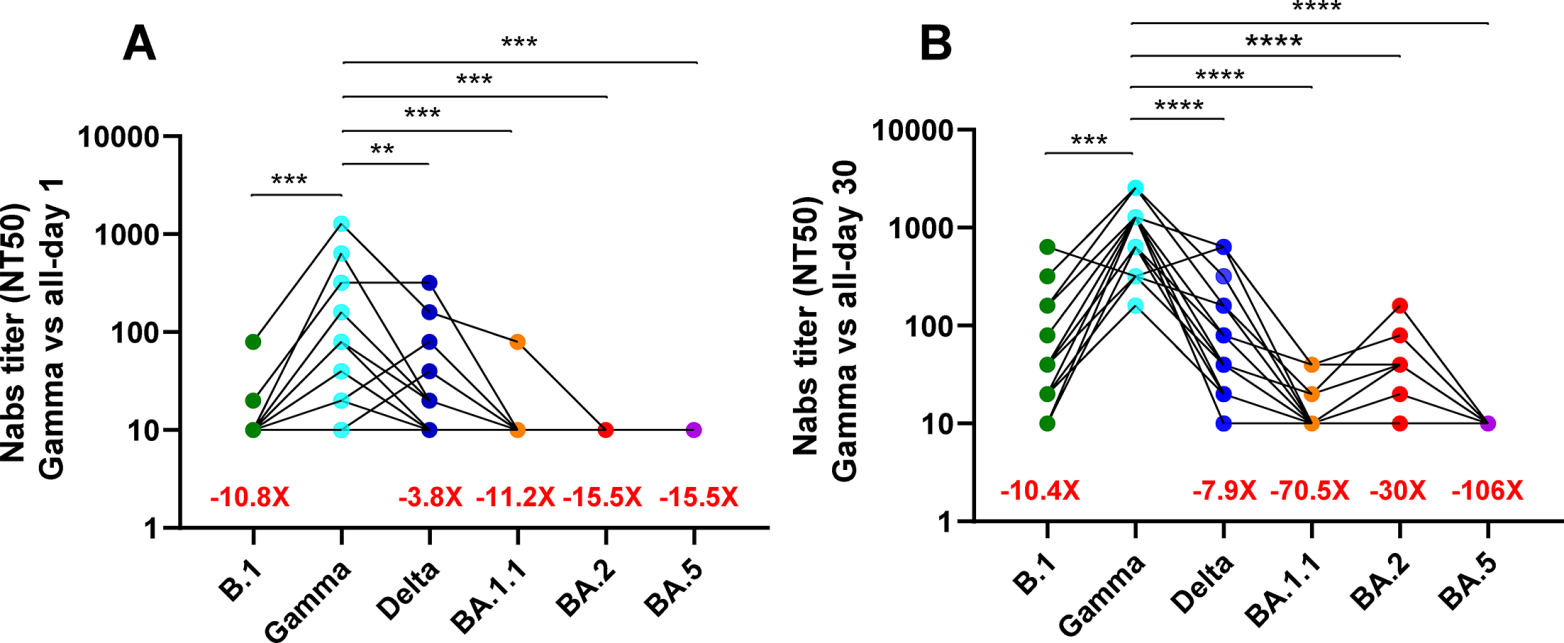
Comparison of neutralizing antibody (Nab) titers in sera obtained from hospitalized patients recruited between May and August 2021. Neutralizing antibody titers were measured in sera collected on day 1 (**A**) (*n* = 20) and day 30 (**B**) (*n* = 20) post-hospitalization against the B.1, Gamma, Delta, BA.1.1, BA.2, and BA.5 strains. These titers were compared to those of the Gamma variant. Each circle represents an individual patient, with different SARS-CoV-2 variants indicated by circles with different colors, as shown on the *x*-axis. Results are expressed as NT50 values on a logarithmic scale. Asterisks denote statistically significant differences between sample groups (**, *P* < 0.01; ***, *P* < 0.001; ****, *P* < 0.0001).

The Nab titers showed significant differences across SARS-CoV-2 variants, particularly when compared to the Gamma variant, which served as the reference. Serum samples from day 1 post-hospitalization exhibited markedly lower Nab titers against Omicron subvariants, with an approximate 15.5-fold reduction compared to Gamma (Fig. 2A). In contrast, reactivity against the B.1 and Delta variants was considerably less diminished, showing 10.8- and 3.8-fold reductions, respectively (Fig. 2A). By day 30, sera collected from the same patients during their convalescent phase (following Gamma or Lambda infection) demonstrated a substantially improved neutralizing capacity against pre-Omicron subvariants compared to day 1 measurements (Fig. 2B). Furthermore, while these day 30 sera showed moderate Nab titers against the BA.2 Omicron subvariants, suggesting enhanced affinity over time, there was no significant difference in the humoral response to the BA.1.1 and BA.5 Omicron subvariants between day 1 and day 30 samples (Fig. 1D and F).

**Fig 3:**
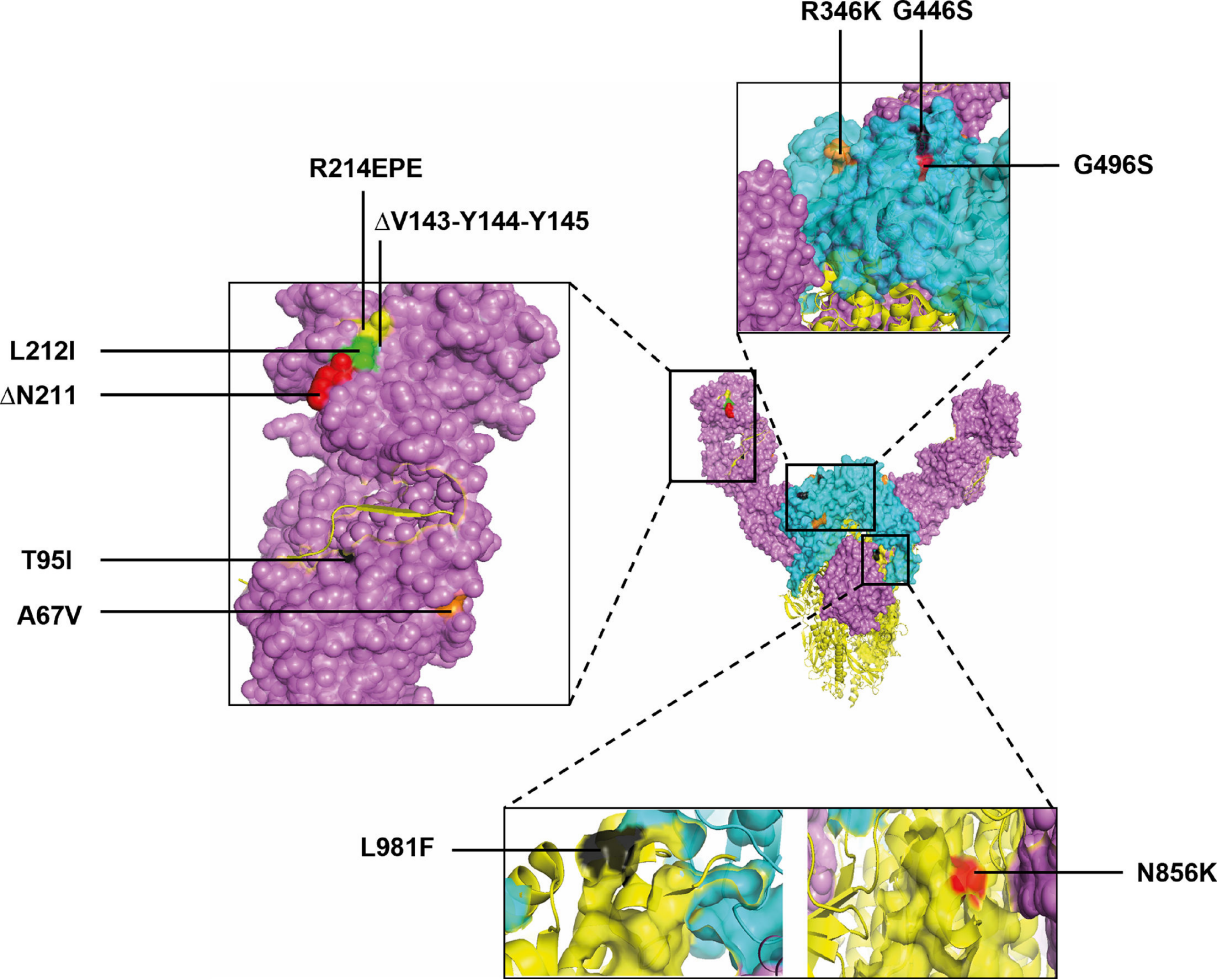
Localization of the amino acid modifications present in the BA.1.1 strain that are not shared with the B.1, Delta, Gamma, BA.2, and BA.5 strains utilized to determine the Nab levels in the sera samples. The structures of the N-terminal domain and receptor-binding domain in the Spike structure (7ZH1, pdb_00007zh1) are in magenta and light blue, respectively, and the localization of the residues analyzed is highlighted in different colors.

To identify the possible reasons for the weak neutralizing response of these sera against the BA.1.1 strain, especially in comparison to the Gamma strain, we examined the amino acid sequences of the Spike proteins from the SARS-CoV-2 variants used in the tests. Among the 38 mutations found in the BA.1.1 variant (relative to the ancestral B.1 strain), we identified 13 unique changes (6 substitutions, 4 deletions, and 3 insertions) that were not present in the Delta, Gamma, BA.2, or BA.5 variants (see Tables S1 and S2 at https://doi.org/10.6084/m9.figshare.30946484). Based on previous research on these mutations, 11 of the 13 have been linked, either directly or indirectly, to immune evasion (Fig. 3; also see Table S3 at https://doi.org/10.6084/m9.figshare.30946484). These mutations may partially explain the low neutralizing response against BA.1.1 observed in sera from patients in the first cohort.

**Fig 4:**
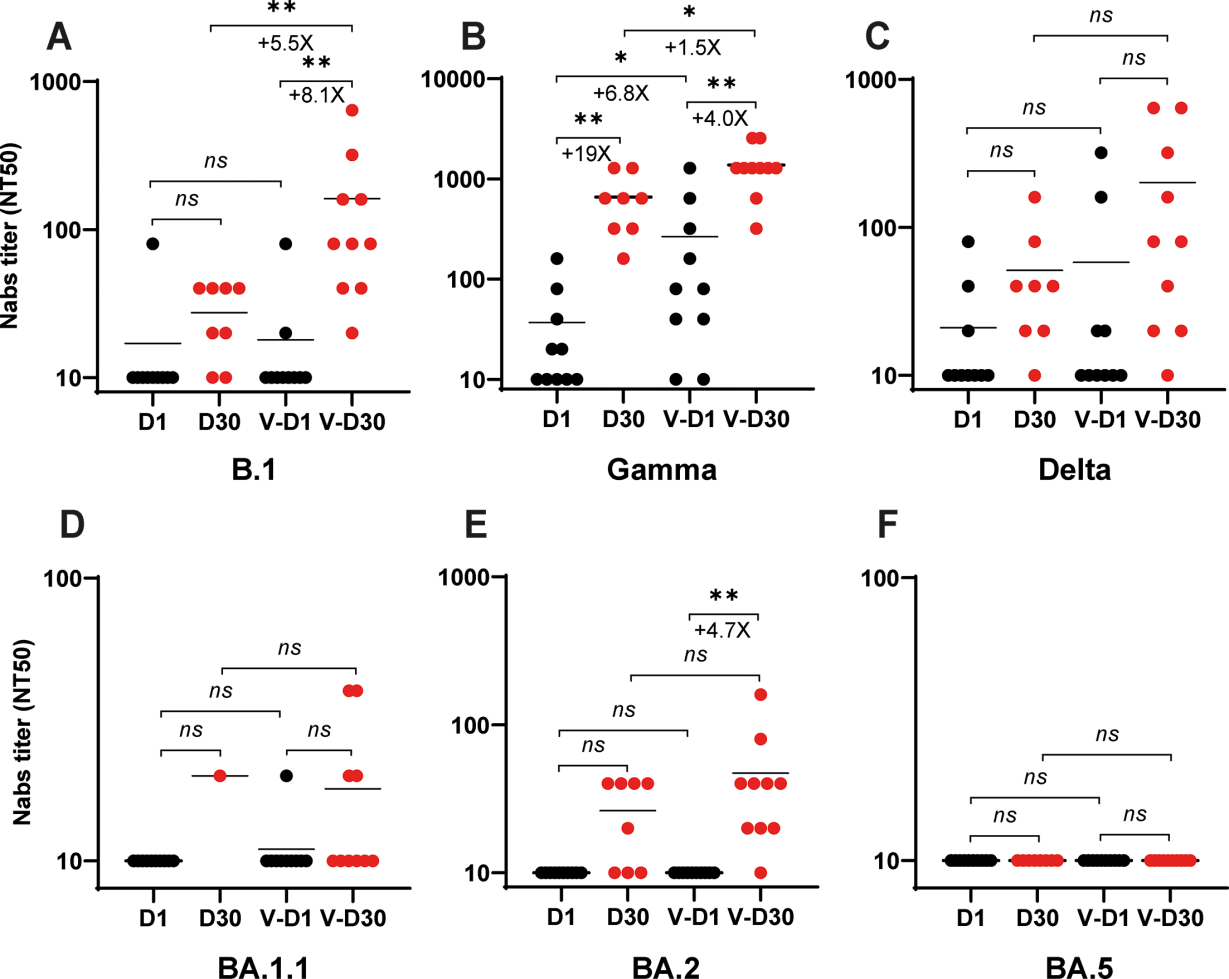
Effect of partial vaccination on the Nab levels in hospitalized COVID-19 patients recruited between May and August 2021. The Nab titers were assessed against pre-Omicron strains, including B.1 (**A**), Gamma (**B**), and Delta (**C**), as well as Omicron variants, such as BA.1.1 (**D**), BA.2 (**E**), and BA.5 (**F**). Results are expressed as NT50 values on a logarithmic scale. Each circle represents an individual patient; black circles correspond to sera collected on day 1 post-hospitalization, while red circles represent sera collected on day 30 during the convalescent stage. Vaccinated individuals (who received one dose of either Sputnik, AstraZeneca, Pfizer, Moderna, or CanSino) and non-vaccinated individuals are indicated below the *x*-axis. The fold change in mean nab titers and significant *P* values are denoted below and on the line, respectively. References: D1 and D30 indicate sera collected from non-vaccinated individuals on day 1 and day 30 post-hospitalization, respectively; V-D1 and V-D30 indicate sera collected from vaccinated patients on day 1 and day 30 post-hospitalization, respectively. Asterisks denote statistically significant differences between sample groups (*, *P* < 0.05; **, *P* < 0.01; ns, no significant).

The Argentine COVID-19 vaccination initiative, launched in January 2021, attained an approximate 65% first-dose coverage by August 2021 (Argentina Ministry of Health, https://www.argentina.gob.ar/coronavirus/vacuna/aplicadas). Among the first cohort of COVID-19 patients mentioned above (Fig. 1 and 2), 50% had received a single administration of Sputnik V (human adenovirus vector; Gamaleya Research Institute, Russia), Covishield (chimpanzee adenovirus vector; Oxford–AstraZeneca, United Kingdom–Sweden), or Sinopharm (inactivated virus; Sinopharm, China) vaccine. Serum samples acquired on day 1 post-hospitalization from these partially vaccinated individuals in the first cohort demonstrated neutralizing activity exclusively against the Gamma variant, exhibiting a 10-fold augmentation in neutralization capacity compared to non-vaccinated counterparts (Fig. 4). Given that people were vaccinated with a B.1-like Spike, these findings indicate that the neutralizing response likely reflects immune memory from the vaccination, which was subsequently boosted by the recent Gamma infection. Analysis of convalescent serum samples collected on day 30 revealed that vaccinated patients mounted augmented cross-neutralizing antibody responses against the B.1 variant (+8.1×), Gamma variant (+4.0×) (Fig. 4A and B), and a very low response against the Delta variant with no significant difference between days 1 and 30 (Fig. 4C). We observed a moderate response against the BA.2 subvariant (+4.7×; Fig. 4E). Nevertheless, a notable absence of neutralizing activity was observed against the BA.1.1 and BA.5 subvariants (Fig. 4D and F), thereby suggesting that a single dose of the aforementioned COVID-19 vaccines was inadequate to confer robust protection against the Omicron variant.

### Convalescent sera from Omicron-infected ambulatory adults showed robust neutralizing antibody responses against pre-Omicron and Omicron variants

In alignment with our prior analysis of the hospitalized patient cohort, we evaluated the neutralizing capacity of sera obtained from the second cohort of ambulatory COVID-19 patients. These samples were also acquired at two distinct time points: day 1 and day 30 post-symptom onset. By July 2022, when the collection of samples from this cohort was finalized, comprehensive vaccination coverage was observed within the Argentine population, with 95% having received a primary dose of a COVID-19 vaccine, predominantly Sputnik and Sinopharm. Second and third dose coverages were recorded at 86% and 45%, respectively. The prevailing vaccine supply limitations during this period necessitated the implementation of a heterologous prime-boost strategy for second and third doses, incorporating Sputnik V (33%) and Covishield (15%), which were mentioned before, but also Comirnaty (mRNA vaccine; Pfizer–BioNTech, United Kingdom) (20%), Spikevax (mRNA vaccine; Moderna, USA) (20%), and PakVak (human adenovirus vector; Cansino, China) (12%) vaccines (14).

**Fig 5:**
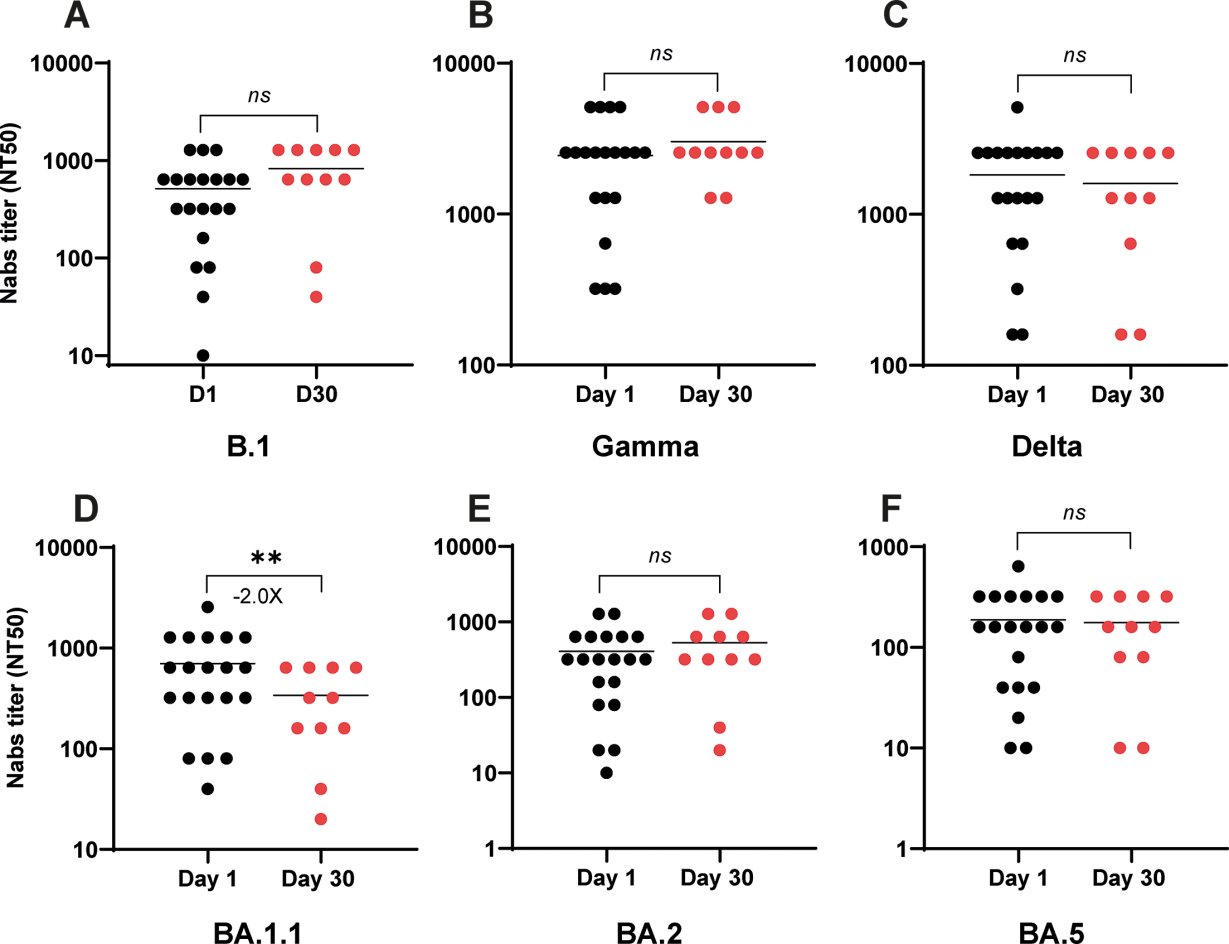
Scatter plots of neutralizing antibody titers against COVID-19 variants. NT50 values increase from day 1 to day 30 post-symptom onset. Pre-Omicron strains B.1 (**A**), Gamma (**B**), and Delta (**C**) show higher neutralization than Omicron variants BA.1.1, (**D**), BA.2 (**E**), and BA.5 (**F**). Asterisks denote statistically significant differences between sample groups ( **, *P* < 0.01; ns, no significant).

The impact of COVID-19 vaccination on ambulatory patients in the second cohort, who had received a minimum of three doses of the SARS-CoV-2 vaccine, was analyzed (Table 1). It is probable that these results were also influenced by the immune response generated from prior natural infections. High levels of NAbs (>400 NT50, Fig. 5) were observed in serum samples collected on day 1 post-symptom onset, particularly against the Gamma variant ($\bar{x}$ = 2,448 NT50) and the Delta variant ($\bar{x}$ = 1,824 NT50). Moderate NAb levels were observed against the BA.1.1 variant ($\bar{x}$ = 702 NT50) and BA.2 ($\bar{x}$ = 410 NT50), while a low response was noted against the BA.5 variant ($\bar{x}$ = 188 NT50) (Fig. 5). When NAb levels in the sera of Omicron-infected patients were compared between days 1 and 30, no significant differences were noted against almost all variants (Fig. 5A through C, and E and F). However, a significant decrease in NAb levels was observed against BA.1.1 (−2.0 lower) (Fig. 5D).

**Fig 6:**
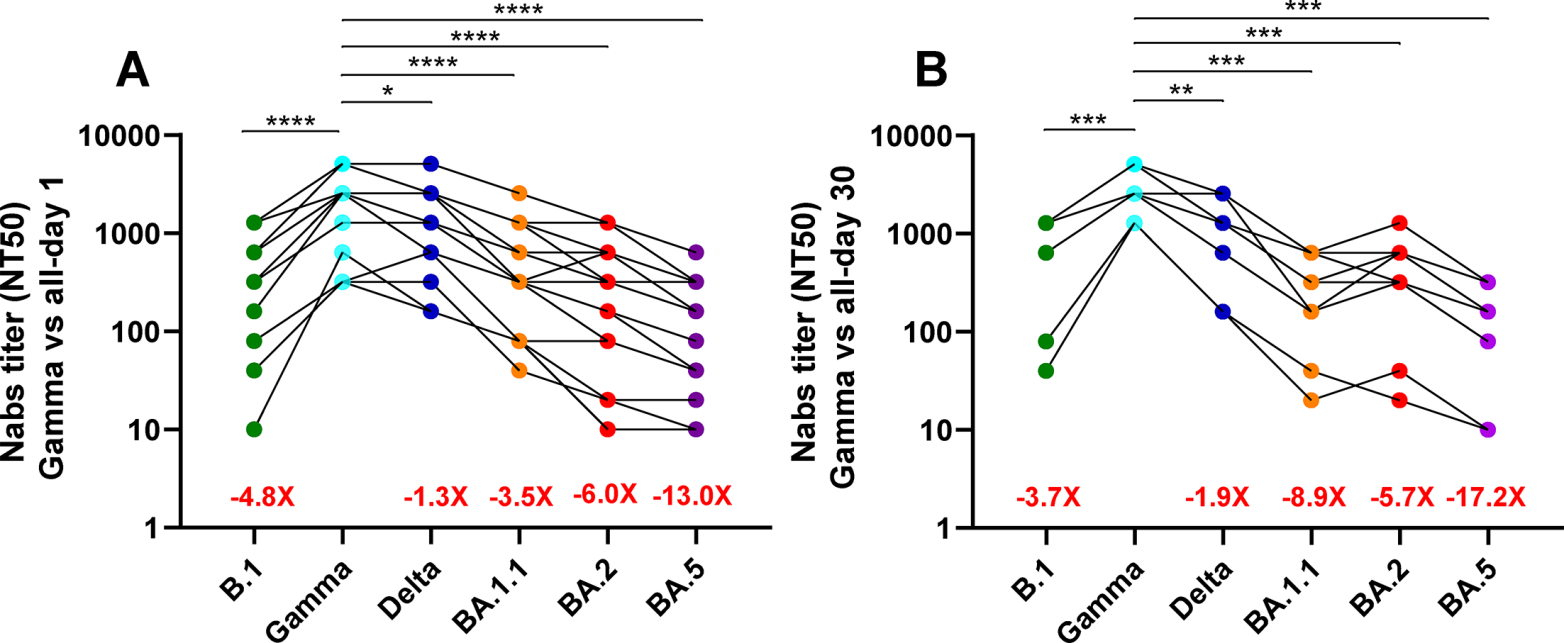
Comparison of Nab titers in sera obtained from ambulatory patients recruited between May and July 2022. Neutralizing antibody titers were measured in sera collected on day 1 (**A**) (*n* = 12) and day 30 (**B**) (*n* = 12) post-symptom onset against the B.1, Gamma, Delta, BA.1.1, BA.2, and BA.5 strains. These titers were compared to those of the Gamma variant. Each circle represents an individual patient, with different SARS-CoV-2 variants indicated by circles of varying colors, as shown on the *x*-axis. Results are expressed as NT50 values on a logarithmic scale. Asterisks denote statistically significant differences between sample groups (*, *P* < 0,05; **, *P* < 0,01; ***, *P* < 0,001; ****, *P* < 0,0001).

As previously indicated for the first cohort, the disparities in NAb levels were particularly evident when the day 30 post-symptom onset data were assessed, with the Gamma variant serving as a reference point due to its highest levels within the panel. A diminished response was noted against the B.1 variant (−3.7×) and the Delta variant (−1.9×); however, the most pronounced differences were recorded against the Omicron subvariants BA.1.1 (−8.9×), BA.2 (−5.7×), and BA.5 (−17.2×) strains (Fig. 6).

## DISCUSSION

The immune response to SARS-CoV-2 infection is significantly affected by various patient characteristics, such as sex, age, prior immunity, and disease severity (15). The severity and outcomes of COVID-19 are predominantly influenced by a patient’s age. Specifically, among adults aged 65 and older, approximately 80% are found to require hospitalization, and a 23-fold increased risk of mortality is experienced by this group when compared to individuals under 65. This variation can be attributed to several factors related to the immune response (16). In the initial cohort of this study, which consisted of patients infected with the Delta or Gamma variants and who were all hospitalized between May and August 2021, 70% of the patients were observed to be aged between 30 and 60 years. Following March 2021, work activities in Argentina were resumed; however, social distancing measures remained recommended until March 2022, particularly for at-risk populations. This apparent discrepancy is likely attributable to the better compliance among older individuals to the various preventive measures, which resulted in their decreased exposure during the second wave of COVID-19. Conversely, the severity of COVID-19 has been significantly correlated with various comorbidities, including obesity, diabetes, hypertension, and respiratory and cardiovascular diseases (16, 17).

In the second cohort, we noted the prevalence of hypothyroidism among COVID-19 women. These findings are consistent with earlier research that established a connection between the vulnerability to severe COVID-19 and thyroid disorders such as hypothyroidism and hyperthyroidism (18). This occurrence may be attributed to a redistribution of ACE2 within tissues; ACE2 serves as the receptor for SARS-CoV-2 entry into host cells, and its expression is modulated by the serum levels of thyroid hormones (19).

The Omicron variant, first identified in November 2021 in South Africa (20), became the predominant SARS-CoV-2 variant in Argentina at the beginning of 2022 (14). This displacement, observed across most countries, was accompanied by the highest peak of infections during the COVID-19 pandemic and, in the majority of cases, a decreased immune response against the Omicron variants (4, 21). Interestingly, high titers of NAb against the Omicron variants were reported in individuals previously infected with the Delta variant (7). Of note, a robust immune response to the Delta variant was demonstrated by serum samples obtained from convalescent patients in Luxembourg who were hospitalized with moderate to severe COVID-19 and infected with the B.1 variant between March and July 2020. Acceptable NAb titers against the B.1 variant were deemed in comparison to those against Delta, showing a reduction of 1.8-fold. However, a significant diminution was observed in the immune response against the BA.2, BA.4, and BA.5 variants, with reductions of 5.0-, 4.5-, and 3.2-fold, respectively. Furthermore, a marked decrease in NAb levels was exhibited against the BA.1 variant, with a 45-fold reduction (9).

A substantial 56-fold decrease in the specific antibody response to the Omicron BA.1 variant was revealed in serum samples from COVID-19 patients hospitalized in Italy during the pandemic’s first wave when compared to the wild-type strain (Italy-INM1) (10). Similarly, while the highest levels of NAbs against the Gamma variant were indicated in serum samples from hospitalized patients in Brazil infected with the Gamma variant between March and December 2020, significantly lower neutralizing capacities were noted against the Delta and Omicron variants, with reductions of 15.7- and 20.0-fold, respectively (8). In Argentina, decreased neutralizing activity against the BA.1 Omicron variant was revealed in plasma from unvaccinated children (−12×) and adults (−7×) when compared with the Wuhan and Delta variants, which displayed similar NAb titers. These individuals were infected mainly with the Lambda and Gamma variants (not Delta) between March 2020 and July 2021, and all experienced asymptomatic or mild disease without hospitalization; their serum samples were collected from various provinces, excluding Córdoba (11).

The cross-neutralization ability of sera in relation to pre-Omicron and Omicron variants was assessed and contrasted in this research. Serum samples were obtained from two distinct cohorts of individuals infected with COVID-19: hospitalized patients (50% of whom had received one dose of vaccination) during the period from May to August 2021, which coincided with the second wave of infections, and ambulatory patients (all of whom had received at least three doses of vaccination) during the fourth wave of COVID-19. Within the initial cohort, the Nab titers were found to be low to moderate against the B.1, Gamma, and Delta variants in samples collected on the first day of hospitalization (Fig. 1A through C). However, significantly elevated Nab titers were exhibited in serum samples collected from the same patients 30 days post-hospitalization compared to those obtained on day 1, particularly against the Gamma variant (+7×, Fig. 1B), which was anticipated, given that these patients were infected with the Gamma and Lambda variants. Furthermore, an increase in titers against B.1 and Delta variants was also noted (Fig. 1A through C).

In the analysis differentiating between vaccinated individuals (those who received a single dose) and non-vaccinated subjects (Fig. 4), a heightened immune response was demonstrated by the vaccinated cohort. This observation indicates a degree of efficacy associated with the COVID-19 vaccines administered to these individuals, despite the fact that their vaccination scheme was incomplete. Given that the individuals received vaccination with a Spike protein of a B.1 strain, it is plausible that these patients possessed preexisting immune memory targeting this protein, resulting in an enhanced NAb response during the initial phase of Gamma variant infection. In Argentina, evidence has demonstrated that individuals vaccinated with Sputnik V maintained neutralizing activity against the Gamma variant in the months following immunization (22). Regarding the rapid NAb response observed in vaccinated individuals following Gamma variant infection, a similar immunological profile has been reported in vaccinated subjects. Specifically, these individuals demonstrated NAb titers against the ancestral virus within 3–4 days after the onset of symptoms when infected with the BA.1 or BA.2 variants (23).

In contrast to the pre-Omicron variants, NAb titers against Omicron strains were significantly lower in both vaccinated and non-vaccinated patients. Even though low NAbs titers against the Omicron subvariants were indicated by these findings, which is consistent with reports from various regions worldwide, the neutralizing capacity of convalescent sera from patients infected with the Gamma and Lambda variants exhibited very low cross-reactivity (−70.5×) against BA.1.1. This level is notably lower than other reported NAbs titers, particularly against BA.1, as observed in studies from Argentina (−7×) (11), Brazil (−20×) (8), and other countries (9, 10) (−45× and −56×, respectively). It is widely recognized that one of the factors contributing to the global prevalence of the Omicron variants is their acquisition of mutations on the Spike protein that facilitate evasion of immunity conferred by monoclonal antibody therapies and vaccinations (21). It is hypothesized that the significant decrease in Nab titers against the BA.1.1 variant observed in hospitalized patients can be attributed to their prior natural infections with the Gamma and Lambda variants, which exhibit considerable antigenic divergence from the BA.1.1 isolate utilized in the neutralization assays. In this context, when considering the Nabs elicited by the pre-Omicron variants in these patients, it appears that these variants were antigenically more akin to BA.2 and BA.5 than to BA.1.1.

When comparing the Spike amino acid sequences of these SARS-CoV-2 variants, we focused on mutations in BA.1.1 that were not shared with the other variants. As mentioned, 11 of the 13 modifications have been previously associated with immune escape in SARS-CoV-2, which could explain the very low neutralizing response observed in the sera of patients from the first cohort. It is important to note that certain combinations have been shown to enhance the individual effects on immune escape by altering antigenicity and neutralization, such as T95I (24), A67V (25, 26), L212I (4, 27), ∆N211 (2, 28), R214EPE (29), ∆V143-Y144-Y145 (24, 30, 31), R346K (32), G446S (33–35), G496S (36, 37), N856K (38), and L981F (39).

Mutations in viral surface proteins have been demonstrated to modify the binding affinity of T- and B-cell epitopes to HLA alleles, a relationship that is pivotal as it directly impacts the efficacy of the adaptive immune response (40). For instance, the Spike protein mutation D614G has been shown to decrease binding affinity to HLA-A*02:01 by a factor of 15, thereby substantially altering immune recognition (41). Conversely, HLA alleles exhibit considerable variation both among individuals and across populations, and this genetic heterogeneity substantially influences the efficacy of immune responses elicited by natural infections or vaccination against SARS-CoV-2 (42, 43).

As mentioned, the combination of immune escape mutations in the Spike protein is one of the primary factors that may explain the neutralizing response against BA.1.1. However, it is important to consider the genetic diversity of HLA alleles in the COVID-19 patients studied. In a previous study, we analyzed the evolution of the Omicron variant in our region and identified several mutations with limited global dissemination (prevalence <0.5%) but unexpectedly high prevalence among SARS-CoV-2 genomes from samples obtained in Córdoba. For example, the L212I mutation showed a prevalence of 16% in strains belonging to the BA.1.1 variant (4).

To assess the potential impact of this mutation on T-cell recognition sites, we conducted an *in silico* analysis focusing on HLA alleles prevalent in the Argentinian population (44) with frequencies higher than 5%, including both class I and class II molecules. We observed that the L212I mutation altered certain T-cell epitopes, suggesting that these changes may facilitate immune escape by the BA.1.1 variant (4). Based on these findings, we propose that the markedly low neutralizing response to BA.1.1 results not only from immune escape mutations but also from the genetic diversity of HLA alleles present in our population.

Our study also included an analysis of Nab levels in ambulatory patients infected with the Omicron variant, utilizing serum samples collected between May and July 2022. The observed Nabs titers were a result of both natural infections and a complete vaccination regimen. An enhanced Nabs response against the Omicron variants was exhibited by the convalescent sera; however, these levels were found to be lower in comparison to those against pre-Omicron variants, particularly the Gamma variant. In this context, it is suggested that this immunological profile is primarily a consequence of the vaccination protocol or prior infections with pre-Omicron variants, rather than a result of natural infection with Omicron itself. This observation aligns with findings that indicate subsequent waves of Omicron were less susceptible to neutralizing antibodies generated by vaccines and that the immune response to the Omicron variants was attenuated (45).

Several limitations are acknowledged in this study. First, the findings are confined to the Omicron variants BA.1.1, BA.2, and BA.5, as more recent variants were not incorporated into the analysis. Additionally, the relatively small sample sizes necessitate a cautious interpretation of the data concerning age, gender, comorbidities, disease severity, and their relationship with the immune response. Furthermore, it is noteworthy that children were not encompassed in either cohort, nor were ambulatory patients included in the first cohort or hospitalized patients in the second cohort. In conclusion, our study further supports the notion that the BA.1.1, BA.2, and BA.5 variants exhibit a capacity to evade NAbs produced as a result of prior infections with the Gamma and Lambda variants in hospitalized patients suffering from moderate to severe COVID-19. Notably, the degree of immune evasion was significantly greater in the BA.1.1 variant when compared to findings from other studies involving patients previously infected with pre-Omicron variants as noted above. Nevertheless, we posit that these results enhance our understanding of the immune response in adults hospitalized with moderate to severe COVID-19. Additionally, this study further advocates for the implementation of epidemiological surveillance programs aimed at monitoring the evolution of SARS-CoV-2, along with the investigation of the role of NAb levels in the neutralization capacity of novel SARS-CoV-2 variants. This kind of research endeavor, similar to the Global Influenza Surveillance and Response System, should facilitate the development of more effective vaccines to address the emergence of new SARS-CoV-2 variants that may circumvent the existing immune responses across diverse global populations.

## MATERIALS AND METHODS

### Sample collection

The first cohort included 40 serum samples from 20 hospitalized COVID-19 patients at HPU, collected between May and August 2021. For these patients, we took 20 samples on day 1 post-hospitalization and another 20 samples on day 30 post-hospitalization. The second cohort consisted of 24 serum samples from 12 ambulatory COVID-19 patients collected from RF between May and July 2022. Similar to the first cohort, 12 samples were taken on day 1 post-symptom onset and 12 on day 30 post-symptom onset. All serum and nasopharyngeal swab specimens were stored at −80°C until use.

### RNA purification and detection by RT-PCR

The RNA purification and detection using RT-PCR methods were performed as previously described (4). Briefly, RNA samples were extracted from nasopharyngeal swabs using the Highway DNA/RNA Puri-Prep-VIRUS Kit (K1501), following the manufacturer’s instructions (46). To determine the levels of SARS-CoV-2 RNA, we utilized the DisCoVery SARS-CoV-2 RT-PCR Detection Kit Rox (DV101, TransGen Biotech Co.). This kit targeted the conserved regions of the Orf1ab and N genes for primer and probe binding sites.

### Viruses and cells

Vero-E6-TMPRSS2 cells (VeroE6 cells overexpressing human TMPRSS2) were cultured in complete media consisting of Dulbecco’s Modified Eagle Medium supplemented with 10% fetal bovine serum (Gibco, Thermo Fisher Scientific), 1 mM glutamine (Invitrogen, Thermo Fisher Scientific), 1 mM sodium pyruvate (Invitrogen, Thermo Fisher Scientific), 100 U/mL penicillin (Invitrogen, Thermo Fisher Scientific), and 100 μg/mL streptomycin (Invitrogen, Thermo Fisher Scientific). The cells were incubated in a humidified incubator with 5% CO_2_ at 37°C. The SARS-CoV-2 variants were isolated at Johns Hopkins University, and virus stocks were propagated on Vero-E6-TMPRSS2 cells, as previously described (47). The viruses used in this study are an ancestral B.1 lineage (hCoV-19/USA/DC-HP00007/2020, EPI_ISL_434688), a Gamma P.1.17 lineage (hCoV-19/USA/MD-HP03867/2021, EPI_ISL_1468644), a Delta B.1.617.2 (AY.106) lineage (hCoV19/USA/MD/HP05660/2021,EPI_ISL_2331507), an Omicron BA.1.1 (B.1.1.529) lineage (hCoV-19/USA/MD-HP25001-PIDAZNSFBL/2022, EPI_ISL_9245416), an Omicron BA.2 lineage (hCoV-19/USA/MD-HP28891-PIDNYQUYZR/2022, EPI_ISL_11962943), and an Omicron BA.5 (B.1.1.529) lineage (hCoV-19/USA/MD-HP32103-PIDCNSQVGY/2022, EPI_ISL_15013106).

### Virus neutralization assay

After performing twofold dilutions of plasma (ranging from 1:20 to 1:2,560), infectious virus was added to the serial dilutions at a final concentration of 1 × 10^3^ TCID_50_/mL and incubated for 1 hour at room temperature. One hundred microliters of the virus-serum mixture (containing 100 TCID_50_ units) was transferred to a 96-well plate containing Vero E6-TMPRSS2 cells in sextuplets and incubated until a cytopathic effect was observed in the controls and the highest serum dilutions. The cells were then fixed and stained, and the neutralization titers were calculated as the highest serum dilutions that eliminated the cytopathic effect in 50% of the wells (NT50).

### Virus sequencing

The SARS-CoV-2 variants were identified through the sequencing of viral genomes. For the SARS-CoV-2 isolates obtained from the first patient cohort (May–August 2021), the genomes were sequenced using the Illumina protocol as described (12). Briefly, total RNA extracted from nasopharyngeal swab specimens underwent complementary DNA synthesis with random hexamers using ProtoScript II (New England Biolabs, E6560), followed by whole-genome amplification with custom-designed tiling primers. Library preparation was performed using the Nextera XT DNA Sample Preparation Kit (Illumina, FC-131-1096). The Nextera XT libraries were sequenced in a paired-end 2 × 150 nt run format on the Illumina MiSeq platform. For the nasopharyngeal samples collected between May and July 2022, the viral genomes were sequenced using the Nanopore protocol, as described (4), following the ARTIC protocol v.3 available online (https://www.protocols.io/view/ncov-2019-sequencing-protocol-v3-locost-bp2l6n26rgqe/v3). The DNA sequencing process was monitored using the MinKNOW software provided by Oxford Nanopore Technologies (48). Data analysis was conducted using a pipeline based on the Epi2me platform (https://epi2me.nanoporetech.com/) developed by Metrichor Ltd., version 2022.04.26-13521, which integrates ARTIC and Pangolin subsections (Artic + Pangolin v.3.3.1). The ARTIC software was utilized to assess the depth of coverage for each bar-coded sample, enabling the exploration of individual amplicons that may not have been adequately amplified using the two primer pools. We analyzed genomes that were 60%–90% complete in terms of DNA sequence and had a coverage greater than 10×. Pangolin was employed to determine the lineage of each sample.

### Statistical analyses

The normal distribution of the data were analyzed using the Kolmogorov–Smirnov test for normality (https://www.socscistatistics.com/tests/kolmogorov/default.aspx). For paired samples with non-parametric distribution, we employed the Wilcoxon matched-pairs signed-rank test using GraphPad Prism 10. A *P* value of less than 0.05 was considered statistically significant (*, *P* < 0.05; **, *P* < 0.01; ***, *P* < 0.001). For the comparison of proportions, we utilized MedCalc (MedCalc 2025), which implements the *N* − 1 chi-squared test as recommended by Campbell (49) and Richardson (50).

## Data Availability

All the complete SARS-CoV-2 genomes generated and presented in this study are publicly accessible through both the GenBank platform (OP302810–OP302821) and the GISAID platform (hCoV-19/Argentina/CBA CIBICI-21/2022–hCoV-19/Argentina/CBA-CIBICI-40/2022).
